# Differences in Gaze Fixation Location and Duration Between Resident and Fellowship Sonographers Interpreting a Focused Assessment With Sonography in Trauma

**DOI:** 10.1002/aet2.10439

**Published:** 2020-02-28

**Authors:** Colin R. Bell, Adam Szulewski, Melanie Walker, Conor McKaigney, Graeme Ross, Louise Rang, Joseph Newbigging, John Kendall

**Affiliations:** ^1^ Department of Emergency Medicine Kingston Health Sciences Centre Kingston Ontario; ^2^ Queen’s University School of Medicine Kingston Ontario; ^3^ Department of Emergency Medicine South Health, Alberta Health Services and Cumming School of Medicine at the University of Calgary Calgary Alberta Canada; ^4^ Department of Emergency Medicine Denver Health Medical Center and University of Colorado Medical School Denver CO

## Abstract

**Objectives:**

We quantified the gaze fixation duration of resident and fellowship sonographers interpreting a prerecorded focused assessment with sonography in trauma (FAST). We hypothesized that all sonographers would fixate on each relevant anatomic relationship but that the duration of fixation would differ.

**Methods:**

We conducted a cross‐sectional study collecting and analyzing the gaze fixations of a convenience sample of current resident and fellowship sonographers. All sonographers viewed a standardized FAST video, and their gaze fixations were recorded using a Tobii X3‐120 eye‐tracking bar. Gaze fixations over nine anatomic regions of interest (ROIs) were identified. These were assessed for normality and analyzed using the Wilcoxon rank sum test at an alpha of 0.05 and Bonferroni correction p value of <0.0034. The chi‐square test and Pearson’s correlation were performed to assess statistical association.

**Results:**

The gaze fixation recordings of 24 resident and eight fellowship sonographers were suitable for analysis. Fourteen of the 24 resident sonographers viewed all ROIs in the FAST, whereas all eight fellowship sonographers viewed each of the nine relevant ROIs. Five ROIs were identified over which at least one resident sonographer did not have a gaze fixation. No statistically significant difference was identified between groups. Resident sonographers gaze fixated over the left upper quadrant (LUQ) splenorenal interface for a median (interquartile range) of 10.64 (9.73–11.60) seconds. The fellowship group viewed the same ROI for 8.43 (6.64–8.95) seconds (p < 0.003). All participants viewed this ROI. No other ROIs had a statistical difference.

**Conclusion:**

Five ROIs were identified that were not visually interrogated by all resident sonographers. Only 14 of 24 resident sonographers visually interrogated every area in the FAST, whereas all fellowship sonographers interrogated every ROI. A statistically significant difference was found in gaze fixation duration between resident and fellowship sonographers in one ROI. Further study is required for gaze fixation assessment to become a tool for the interpretation component of point‐of‐care ultrasound.

Point‐of‐care ultrasound (POCUS) and the focused assessment with sonography in trauma (FAST) examination are core competencies of emergency medicine (EM) training^1^, ^2^, ^3^ and part of the Advanced Trauma Life Support approach to trauma assessment.^4^ The EM Milestones and traditional credentialing of FAST have been based on performing a fixed number of scans, direct observation, and written examinations.^1^, ^2^, ^3^, ^5^ Furthermore, POCUS education, assessment, and supervision are variable among EM residency training programs,^5^ so even if learners completed the requisite number of studies, their proficiency is unlikely to be uniform. As EM education transitions to a competency‐based curriculum, assessment must evolve from numeric mandates toward more objective proficiency assessment methods. Alternatives that have been explored include direct observation, simulation, checklists, global rating scales, or hand motion assessment.^6^, ^7^, ^8^, ^9^ Each is time‐consuming, resource‐intensive, or expensive or has not been validated. Accordingly, none of these assessment methods are in widespread use, so there is no standardization in determining POCUS proficiency for ultrasound learners. As POCUS permeates medicine and competency‐based assessments are mandated, it is imperative that robust skill assessment methods are developed utilizing technology that has the capacity to be automated.

Procedural skills are integral to the practice of medicine across multiple specialties. In 1997, Kovacs adapted the closed‐loop theory for the learning of procedural skills.^10^ Sensory inputs perceived during a motor act, when interpreted with knowledge of the outcome, should result in a further refinement in technique, leading to subsequent improvement in skill performance. While performing a POCUS examination, the physician operator is near simultaneously generating an image and interpreting it. This complex interplay of image acquisition and interpretation represents a “real‐time” cycle of the Kovac’s adapted closed‐loop theory.^10^ When assessing POCUS proficiency, it may be difficult for the operator or assessor to determine which component of this rapid cycle failed when the examination is inadequately performed.

Objective proficiency assessment methods for the image generation component of the FAST examination have been previously studied.^8^, ^9^, ^11^ However, objective assessment of the interpretation component of POCUS remains informal in many cases.^12^ Since the vast majority of FAST examinations will be free of pathology, a determination that a study is “negative” does not entirely assess whether an individual is competent in the interpretation component of the examination. An additional element in assessing interpretation competency is an operator’s ability to visualize and assess the critical areas of an image or study, regardless of whether pathology is present or absent. A further challenge is the ability to analyze critical regions of studies in real time. When a FAST examination is being performed, multiple regions critical to the interpretation of an image may appear on screen nearly simultaneously. The issue of interpretive accuracy is further complicated by a lack of methods to objectively ensure that a POCUS operator has visually interrogated all critical anatomic regions of interest (ROIs) prior to rendering an interpretation.

Eye‐tracking technology has the ability to show the locations, durations, and order of gaze fixation on aspects of an image. The gaze fixation behaviors of subjects have been demonstrated to be associated with level of expertise across multiple skills in many domains.^13^, ^14^ In medicine, assessment of eye fixation tracking has been performed using static images for diagnosis^15^, ^16^, ^17^, ^18^ or dynamic images for specific targeted evaluations.^19^, ^20^, ^21^, ^22^, ^23^ At present, very little is known about the eye fixations of POCUS operators interpreting dynamic images with multiple near simultaneously appearing critical anatomic relationships, such as those generated during FAST. In an effort to generate new hypotheses, we set out to quantify and assess the gaze fixations of resident and fellowship sonographers as they interpreted a FAST. We hypothesized that both resident and fellowship sonographers would have a receptive gaze fixation over each ROI in the FAST, but that the locations and durations of their respective gaze fixations would differ.

## METHODS

### Study Design and Participants

We conducted a cross‐sectional study collecting and analyzing the gaze fixations of a convenience sample of 25 current resident sonographers with eight fellowship sonographers who had either completed (five) or were in the process of completing (three) an ultrasound fellowship. All resident participants were in the process of completing residency at the time of enrollment. Of the group of current resident sonographers, one had not met the American College of Emergency Physicians (ACEP) minimum requirement of performing at least 25 FAST examinations, and five had not completed 150 or more basic POCUS studies consistent with ACEP guidelines prior to enrollment.^1^ All fellowship sonographers were actively engaged in quality improvement at one of two North American teaching hospitals. The Colorado Multiple Institutional Review Board (No. 16‐1590) and the Queen’s University Health Science Research Ethics Board (No. 6023366) approved the study. Informed consent was obtained from all participants in the study. Participation was voluntary and no compensation was provided. There were no specific exclusion criteria.

### Stimulus Image Creation

Data were collected during a single session at each location. A continuous video of a complete FAST examination recorded on a single live trauma patient with a computed tomography (CT) abdomen negative for intraperitoneal free fluid or solid organ injury was recorded in a deliberate fashion. The video was edited, eliminating sections that may have been unclear, rapid, or excessively redundant using iMovie (Apple Inc.). Anatomic structures predictably moved across the screen in a linear, controlled, and consistent manner. Each quadrant was prefaced with an appropriate 4‐second title as an internal control to confirm eye tracker calibration.

### Area and ROI Creation and Gaze Fixation Mapping

The edited video segments of the FAST were transferred into the Tobii Pro Lab Software (Tobii Technology). The total video duration was 1 minute 48 seconds including titles. Areas of interest (AOIs) were created within the Tobii Pro Lab software to collect information on the participants’ gaze fixations over the specific anatomic ROIs as described in the SDOTs of supplement 4 of Lewiss et al.^6^ as well as both diaphragms consistent with other studies.^24^ These AOIs moved dynamically with the anatomic ROI within each segment as the video played. If a particular ROI disappeared from the video frame before returning, multiple AOIs were created within the Tobii Pro Lab software corresponding to the particular ROI to capture all gaze fixations occurring during each moment an ROI was visible to the participant. The AOIs corresponding to each ROI were created by primary author CB and subsequently verified by senior author JK prior to data collection.

### Data Collection

Each data collection session took place in a darkened room. A standard clinical vignette describing the video was read, and participants were asked to view the entire video before rendering an interpretation on the outcome of the examination. All participants were seated approximately 65 cm from a 1920 × 1080 resolution, 15.6‐inch screen with an attached Tobii Pro X3‐120 eye‐tracking bar calibrated to the screen. After a participant‐specific visual calibration procedure, the standardized video was played in real time. Participants were not able to view any part of the video more than once. Eye motions and gaze fixations were recorded for analysis. At the conclusion of the video all participants were asked to interpret the examination as either positive or negative for intraperitoneal free fluid.

### Data Display

Heat maps and gaze plots were created using the Tobii Pro Lab software to display the AOIs and the fixation patterns. Heat maps use the fixation data points without order. The different colors represent the time spent fixated at a particular AOI. A gaze plot displays individual fixations that are sequentially numbered fixations. The size of the shaded region surrounding the gaze fixation corresponds to the duration the subject focused on the area.

### Data Analysis

The recorded gaze fixations of 24 resident and eight fellowship sonographers were suitable for analysis. These gaze fixation recordings were analyzed using the Tobii Pro Lab software and exported using the default settings of the fixation filter.^25^ Data were imported into Stata 13.1 (StataCorp LLC) for statistical analysis. AOIs corresponding to the same ROI underwent a planned post hoc aggregation for analysis. Medians and interquartile ranges (IQRs) were calculated for each anatomic relationship for both AOIs and ROIs. The data were assessed for normality and analyzed using the Wilcoxon rank sum test at an alpha of 0.05. A Bonferroni correction was used to account for multiple comparisons; results were considered significant at a p value of <0.0034. Chi‐square test was performed to analyze the number of ROIs viewed between each group. Pearson’s correlation was calculated between the ROIs viewed and the demographics of the participants. One resident sonographer was not included in the gaze fixation analysis as the eye tracker had lost visual calibration during her data collection.

## RESULTS

**Figure 1:**
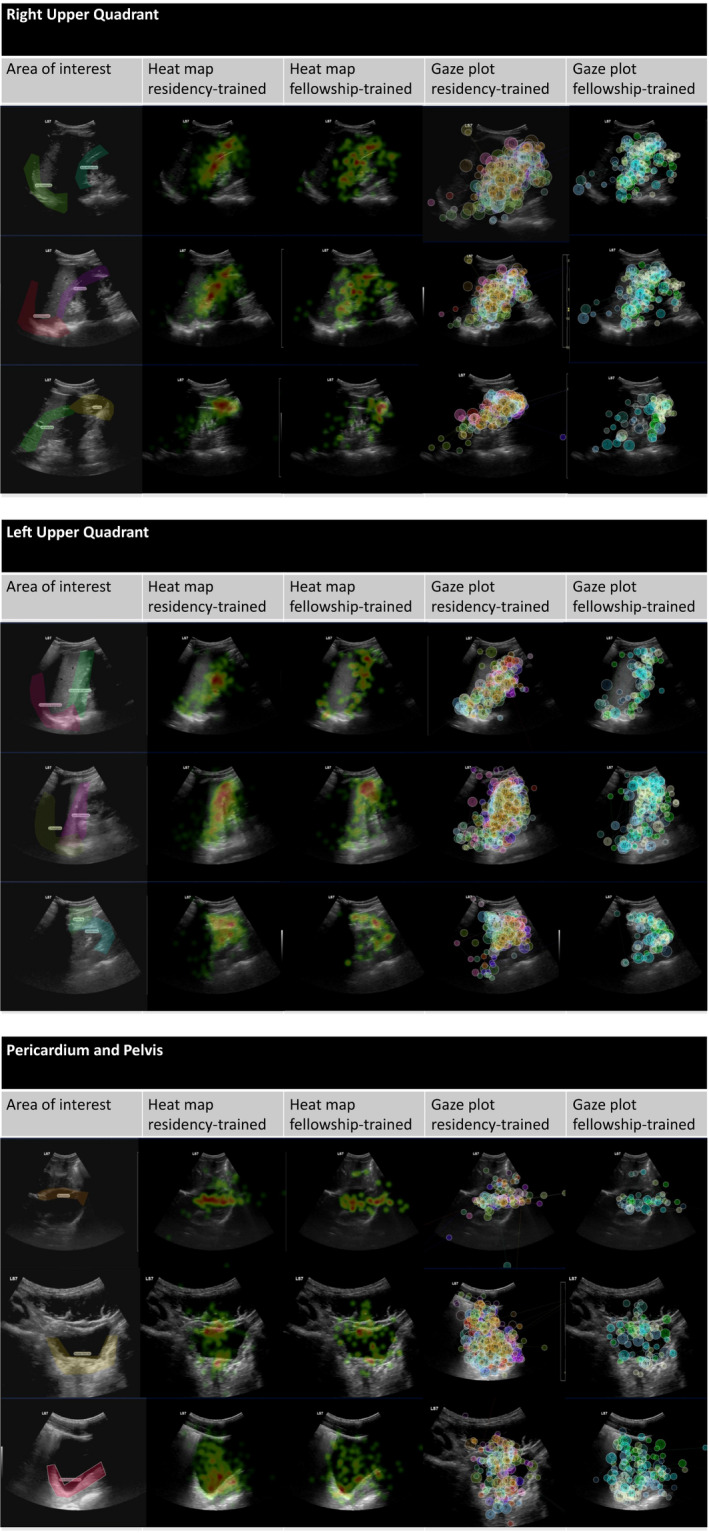
Areas of interest (AOI) and comparison of heat map and gaze plot between resident and fellowship sonographers at FAST examination locations. AOIs are the predetermined anatomic locations scrutinized as part of a FAST examination. Heat maps use the fixation data points without order. Different colors represent different intensities of the fixations. Green represents a smaller number of fixations and red represents the most intense regions of fixation. Gaze plots are an alternative method to assess gaze fixation with the size of the bubble representing the duration of the fixation at a particular location. FAST = focused assessment with sonography in trauma.

Fifteen unique AOIs were created over the video segments a priori corresponding to nine different ROIs visually interrogated during the FAST (Figure 1). All AOIs were analyzed individually and subsequently underwent planned post hoc aggregation into nine ROIs for analysis. Representative heat maps and gaze plots were created for each video segment for both groups of participants. The demographics of both groups of sonographers are presented in Table 1.

**Table 1:** Demographics of the Resident and Fellowship Sonographers Analyzed in the Study

Variable	Resident	Fellowship
PGY‐1	5 (20)	
PGY‐2	4 (16)	
PGY‐3	4 (16)	
PGY‐3 CCFP‐EM*	7 (28)	
PGY‐4	2 (8)	
PGY‐5	3 (12)	
Staff emergency physician		8 (100)
POCUS fellowship in progress		3 (38)
POCUS fellowship completed		5 (63)
Median FAST no	103	>500
Interquartile range FAST	55–197	
Median total POCUS studies performed	308	>2,000
IQR total	228–588	
Subjects who had completed EM residency program training requirements for POCUS	21 (88)	8 (100)
Subjects residents who had completed the minimum of 25 supervised FASTs	24 (95)	8 (100)
Subjects who had completed a minimum of 150 basic POCUS studies with 25 minimum in each area	22 (88)	8 (100)
Primary site Queen's University, Kingston, Ontario, Canada	25 (100)	3 (38)
Primary site Denver Health Medical Center, Denver, CO		5 (63)

Data are reported as *n* (%).

FAST = focused assessment with sonography in trauma; POCUS = point‐of‐care ultrasound.

* Denotes training level of the single resident sonographer excluded from gaze fixation analysis.

### ROI Analysis

Areas of interest in different video segments representing the same anatomic relationship within each quadrant were combined into nine ROIs. Median gaze fixation durations, IQRs, and the number of participants viewing each ROI for the grouped resident and fellowship sonographers are presented in Table 2.

**Table 2:** ROI Fixation Times of Resident and Fellowship Sonographers

ROI	Resident			Fellowship			p‐value
	Median (sec)	IQR	Number viewed area (%)	Median (sec)	IQR	Number viewed area (%)	
RUQ diaphragm	0.97	0.54–1.74	22 (92)	1.54	1.24–2.41	8 (100)	0.06
RUQ hepatorenal interface	5.53	4.41–6.15	24 (100)	4.41	3.38–5.01	8 (100)	0.11
RUQ liver tip and inferior kidney	2.71	1.71–3.13	22 (92)	1.33	0.88–2.32	8 (100)	0.03
LUQ diaphragm	0.87	0.45–1.53	19 (79)	0.94	0.53–1.39	8 (100)	0.77
LUQ splenorenal interface	10.64	9.73–11.60	24 (100)	8.43	6.64–8.95	8 (100)	<0.01*
LUQ spleen tip and inferior kidney	1.67	1.12–2.87	22 (92)	1.62	0.96–2.28	8 (100)	0.32
Pericardium	8.61	7.05–10.48	24 (100)	5.54	3.90–7.83	8 (100)	0.06
Pelvis transverse	0.95	0.58–2.17	24 (100)	1.05	0.29–1.44	8 (100)	0.54
Pelvis longitudinal	1.90	0.92–2.57	22 (92)	1.31	1.11–2.28	8 (100)	0.59
Total time	31.16	27.42–37.48	22 (92)	25.82	21.33–31.89	8 (100)	0.13

	Median (ROIs viewed)	IQR	Number viewing all ROIs(%)	Median (ROIs viewed)	IQR	Number viewing all ROIs (%)	p value
Total ROIs viewed	9	8–9	14 (58)	9	9–9	8 (100)	0.18

LUQ = left upper quadrant; ROI = region of interest; RUQ = right upper quadrant.

* Statistically significant p < 0.0028.

Fourteen of the 24 analyzed resident sonographers viewed all nine ROIs in the FAST, whereas all fellowship sonographers viewed every ROI. Five ROIs were identified that were not visually interrogated by at least one resident sonographer.

When interpreting the left upper quadrant (LUQ) splenorenal interface, resident sonographers gaze fixated over the ROI for a median (IQR) of 10.64 (9.73–11.60) seconds. Fellowship sonographers’ gaze fixated over the same ROI for a median (IQR) of 8.43 (6.64–8.95) seconds. This was found to be statistically significant (p < 0.0025). All resident and fellowship sonographers analyzed viewed the LUQ splenorenal interface ROI.

On ROI analysis of the gaze fixation duration of the total time, right upper quadrant (RUQ), pericardium, and transverse and longitudinal views of the pelvis, no statistically significant difference between the resident and fellowship sonographers was found.

The chi‐square test and Pearson’s correlation were calculated between and amongst resident and fellowship sonographers and their demographics. The results are presented in Data Supplement S1, Table S1 (available as supporting information in the online version of this paper, which is available at http://onlinelibrary.wiley.com/doi/10.1002/aet2.10439/full). No statistically significant difference was identified.

### Interpretation Analysis

All resident and fellowship sonographers correctly interpreted the FAST examination as negative for the presence of intraperitoneal free fluid. This was congruent with the CT scan from the initial trauma patient.

### Heat Map and Gaze Plot Generation

Heat maps were created to visually display the gaze fixation locations of both groups. Gaze plots were also created to display the order taken by each POCUS operator when analyzing a particular segment of the stimulus. As expected the majority of fixations for both the resident and the fellowship group were within the ROIs in the FAST examination.

## DISCUSSION

In this hypothesis‐generating study, five ROIs in the FAST examination were identified that were not visually interrogated by the resident group. Only 14 of the 24 analyzed residents interrogated every ROI in the FAST, while all fellowship sonographers had at least one gaze fixation within each ROI in the FAST. Furthermore, the gaze fixation duration of resident and fellowship sonographers differed over the LUQ splenorenal interface. The gaze of resident sonographers fixated over the LUQ splenorenal interface for longer than their fellowship counterparts while remaining largely similar over the RUQ, pericardium, and pelvis.

This was a pilot study designed to replicate and assess the image interpretation component of a live FAST examination. We believe that by assessing and analyzing different groups of POCUS operators, we may eventually be able to determine a "criterion standard” of image interpretation. This standard may have future utility for developing an automated objective proficiency assessment for medical care providers using POCUS, in contrast to traditional labor‐intensive approaches presently used for competency assessment.

Sonographers may interrogate a particular anatomic relationship and render an internal opinion prior to moving onto the next ROI, and this may explain why not all AOIs contained a receptive fixation. All of the fellowship sonographers had a gaze fixation within at least one of the AOIs corresponding to an ROI. Of the 24 resident sonographers whose gaze fixation recordings were suitable for analysis, all had met the ACEP minimum requirements for training. However, when interpreting the FAST, 10 resident sonographers did not have a gaze fixation over at least one of the ROIs. Any cognitive processing related to image interpretation begins with visual fixation; this represents a potential clinical pitfall. Although not found to be statistically significant, the absence of a gaze fixation over a particular ROI may be clinically significant because it represents the possibility that sonographer may incorrectly interpret the FAST, thus potentially failing to identify a patient with hemoperitoneum, hemothorax, or other traumatic injury.

The ROIs visually interrogated by all analyzed participants were the RUQ hepatorenal interface, LUQ splenorenal interface, pericardium, and the transverse pelvis. These ROIs are locations where a large volume of free fluid would be likely to be visible and the regions traditionally taught as sensitive for the detection of hemoperitoneum.^26^ The ROIs incompletely interrogated by the resident sonographers were the RUQ and LUQ diaphragms, the liver tip, and spleen tip as well as the longitudinal view of the pelvis. Importantly, the liver tip has been noted to be the most sensitive ROI in the RUQ, and the LUQ diaphragm was the most sensitive region in the LUQ.^24^ Both of these regions are most likely to be representative of the overall result and may reveal pathology when the rest of the quadrant does not.^24^ The RUQ diaphragm, LUQ spleen tip, and inferior kidney ROIs incompletely visualized by the residents have been found to be less sensitive than other ROIs in the FAST.^24^ Our hypothesis was that all resident sonographers would visually interrogate all ROIs.

While it may be possible to attribute the shortcomings of the resident sonographers to their training, the ultrasound faculty at Queen’s University have been faculty at other residency training programs, and are involved in national POCUS training through ACEP, CAEP, CPOCUS, and other POCUS courses. Resident sonographers in the Queen’s EM residency program fulfill the national POCUS training guidelines from CAEP^2^ prior to graduation. These guidelines largely overlap the ACEP guidelines.^1^ With respect to image interpretation, all Queen’s EM residents must successfully complete a POCUS image interpretation test yearly, and all residents are required to complete the ACEP online emergency ultrasound examination once during residency. These requirements are not dissimilar to other North American EM residency training programs^12^ and it is likely that the POCUS training at Queen’s University is not substantially different from other North American EM programs.

The different format of the study may have captured image interpretation behavior that actually occurs in the clinical environment instead of an image interpretation test. The majority of studies obtained during POCUS training are negative for pathology. As a result, the emphasis in training is on obtaining a view of the quadrant rather than exhaustively interpreting it. In this context gaze fixation tracking may have use as a teaching tool demonstrating objectively display where trainees looked while interpreting a POCUS.

When compared with resident sonographers, the gaze fixation durations of the fellowship sonographers were typically within the ROIs for a shorter period of time, although the difference was only statistically significant in the LUQ splenorenal interface. It is possible that these fellowship sonographers may have been more confident when interpreting the FAST compared to resident sonographers, allowing the fellowship sonographers to render an opinion and proceed to interrogate the next ROI. Conversely, when compared to resident sonographers, the fellowship sonographers spent more time fixated on the ROI above the diaphragm on the right side. Several studies have demonstrated POCUS to be less sensitive but highly specific for the detection of solid organ injury, hemothorax, and pneumothorax.^27^, ^28^ Resident sonographers may be spending less effort interpreting the ROIs at and above the diaphragm as well as the spleen tip as these images are more complex to interpret and less sensitive for pathology.^24^

When displayed as a heat map or a gaze plot, the fixations of both groups largely overlapped at the same critical ROIs. It appears that several gaze fixations occurred in the organ parenchyma and above the diaphragm. These fixations were outside the anatomic relationships traditionally interrogated for hemoperitoneum in the FAST.

## LIMITATIONS

Our study had several limitations. As a correlational study with multiple comparisons, the results should be interpreted as hypothesis‐generating to inform the design of future studies. We were sensitive to this issue and included a Bonferroni correction to account for this. Furthermore, we were unable to complete a sample size calculation as we could not identify a representative population performing a similar task. Within the gaze fixation analysis, all resident and fellowship sonographers had obtained the ACEP minimum requirements for interpreting FAST examinations. This similarity may have led to a greater likeness in the gaze fixation patterns of both groups. Additionally, the groups of resident and fellowship sonographers were different in size, and all resident sonographers were from a Queen’s University. Had we chosen comparator groups with greater differences in training, or had a larger group of fellowship sonographers, greater differences in the ROI gaze fixation duration or number of ROIs viewed may have been identified. Ideally, the groups of participants would have been larger, from both sites, and equally sized.

Second, AOI boundaries are clearly defined and it is difficult for the designer to determine the exact boundary between where an interrogator ceases to gain useful information. If the AOI were too generous, fixations that did not result in the acquisition of useful information would have been included; whereas if the AOI was too small fixations, where the participant gathered useful information would have been excluded. Both situations may have led to a greater similarity between groups. Moreover, not all the solid organs were quantified with AOIs and therefore fixations over them could not be analyzed. In an ideal stimulus video, an AOI would correspond to an ROI with every relationship appearing only once. In our video the ROI structures occasionally appeared and disappeared from frame before returning with different dimensions and in new areas on the screen. The reappearance of the ROI mandated the creation of a new AOI within the software. If each ROI only appeared once for the duration of the video, our gaze fixation duration results may have been different, and a significant difference may have been found. If all anatomic structures in each quadrant in addition to the ROIs interrogated during a FAST examination had a corresponding AOIs, it would have been possible to gather gaze fixation data, allowing for a more comprehensive analysis that may have revealed a difference between sonographer groups.

During a FAST examination, multiple AOIs may appear on screen near simultaneously making statistical analysis more complex as different operators may use separate paths to reach the same conclusion. Analyzing the cognitive processes of participants using a mixed‐methods approach by adding a qualitative cognitive task analysis to the experimental design could have addressed this challenge.^29^ Additionally, a comprehensive analysis of spatial statistical methods would have been useful. 30

This study was compartmentalized to the interpretation component of POCUS. Members of both the resident and the fellowship sonographers, if given the opportunity to generate their own images, may have followed a different order. Additionally, if given control over the video, they may have reviewed a segment multiple times or scrutinized a particular frame in greater detail. Members of the fellowship group were actively involved in quality assurance and may have more experience reviewing the images of others thus putting them more at ease during the study.

Finally, as with any study, a Hawthorne effect may have been present, where participants adapted their behavior knowing that they were being studied. Solutions to this problem may be to incorporate the experiment into standard ultrasound quality improvement procedures such as routine image review.

## CONCLUSIONS

Eye‐tracking and gaze fixation assessment may become a promising tool for the evaluation of the interpretation component of point‐of‐care ultrasound. Differences in the gaze fixation behavior between resident and fellowship sonographers were identified for the image interpretation component of the focused assessment with sonography in trauma examination. Only 14 of the 24 analyzed resident sonographers visually interrogated every area in the focused assessment with sonography in trauma, whereas all fellowship sonographers interrogated every region of interest. Five regions of interest were identified that were not visually interrogated by all resident sonographers, which included some of the most sensitive areas of the focused assessment with sonography in trauma. Resident sonographers fixated for more time on the left upper quadrant splenorenal interface, when compared to fellowship sonographers. Current eye‐tracking analysis methods have some use for interpreting dynamic stimulus such as a point‐of‐care ultrasound study. The future development of new assessment techniques involving the gaze fixations of point‐of‐care ultrasound operators may become a promising method for objective proficiency assessment. Additional studies with heterogeneous groups of participants, diverse point‐of‐care ultrasound types, and images with varied pathology are needed to transform gaze fixation tracking into an objective proficiency assessment method.

## Supporting information


**Data Supplement S1.** Supplemental material.
