# Supplementary material for: Differences in Gaze Fixation Location and Duration Between Resident and Fellowship Sonographers Interpreting a Focused Assessment With Sonography in Trauma
Source: AEM Educ Train. 2020 Feb 28;5(1):28–36. doi: 10.1002/aet2.10439 (PMC7821074; doi:10.1002/aet2.10439)
Supplement: Supplementary file 1 — Data Supplement S1. Supplemental material. [file AET2-5-28-s001.pdf]

# Data Supplement S1. Supplemental material

Table S1: Resident and Fellowship sonographers interpreting AOIs corresponding to ROIs.

| | Area of interest analysis | | | | | | | Region of interest analysis |
|---|---|---|---|---|---|---|---|---|
| | Residency-trained | | | Fellowship-trained | | | | |
| AOI | Median (sec) | IQR | Number viewed area (%) | Median (sec) | IQR | Number viewed area (%) | p value | Corresponding ROI |
| RUQ diaphragm 1 | 0.59 | 0.36-1.57 | 8 (33) | 0.58 | 0.44-0.82 | 7 (88) | 0.95 | RUQ diaphragm |
| RUQ diaphragm 2 | 0.75 | 0.40-1.49 | 22 (92) | 1.19 | 0.86-1.52 | 8 (100) | 0.19 | |
| Hepatorenal interface 1 | 2.35 | 1.83-3.70 | 24 (100) | 1.57 | 1.25-3.00 | 8 (100) | 0.13 | RUQ hepatorenal interface |
| Hepatorenal Interface 2 | 0.35 | 0.32-0.40 | 4 (17) | 0.39 | 0.33-0.44 | 2 (25) | 1.00 | |
| Hepatorenal interface 3 | 2.95 | 2.27-3.04 | 24 (100) | 2.44 | 1.73-2.97 | 8 (100) | 0.16 | |
| Liver tip and inferior kidney | 2.71 | 1.71-3.05 | 24 (100) | 1.33 | 0.92-2.06 | 8 (100) | 0.03 | RUQ liver tip and inferior kidney |
| LUQ diaphragm 1 | 0.40 | 0.27-0.61 | 11 (46) | 0.73 | 0.35-0.93 | 7 (88) | 0.53 | LUQ diaphragm |
| LUQ diaphragm 2 | 0.56 | 0.40-0.73 | 15 (63) | 0.45 | 0.42-0.63 | 7 (88) | 0.56 | |
| Spleen kidney interface 1 | 8.76 | 7.72-9.72 | 24 (100) | 6.11 | 5.04-7.76 | 8 (100) | <0.01 | LUQ splenorenal interface |
| Spleen kidney interface 2 | 1.84 | 1.10-2.80 | 24 (100) | 1.86 | 1.57-1.98 | 8 (100) | 0.94 | |
| Spleen tip | 0.42 | 0.29-0.69 | 15 (63) | 0.44 | 0.44-0.44 | 3 (38) | 0.86 | LUQ spleen tip and inferior kidney |
| Left kidney inferior pole | 1.53 | 0.81-2.62 | 22 (92) | 1.40 | 0.84-1.93 | 8 (100) | 0.58 | |
| Pericardium | 8.34 | 7.00-10.51 | 24 (100) | 5.54 | 4.03-7.24 | 8 (100) | 0.09 | Pericardium |
| Pelvis transverse | 0.95 | 0.60-2.37 | 20 (83) | 1.05 | 0.29-1.36 | 8 (100) | 0.54 | Pelvis transverse |
| Pelvis longitudinal | 1.69 | 0.84-3.13 | 23 (96) | 1.30 | 1.13-1.88 | 8 (100) | 0.73 | Pelvis longitudinal |

Table S2 Statistical associations between ROIs viewed and resident and fellowship-sonographers demographics

| Comparison | Statistical test | Result | p-value |
|---|---|---|---|
| Resident and fellowship- sonographers by ROIs viewed | Chi-square | 4.85 | 0.18 |
| Resident sonographers by Royal College of Physicians or Canadian College of Family Physician EM training stream | Chi-square | 3.30 | 0.34 |
| Resident sonographers by PGY group | Chi-square | 16.90 | 0.32 |
| Resident and fellowship sonographer and number of FASTs performed | Pearson's correlation | 0.11 | 1.00 |
| Resident and fellowship sonographer and total number of POCUS performed | Pearson's correlation | 0.15 | 1.00 |
| Resident sonographers and total number of FASTs performed | Pearson's correlation | -0.27 | 0.59 |
| Resident sonographers and total number of POCUS studies performed | Pearson's correlation | -0.29 | 0.51 |
